# The Impact of Prompts and Feedback on the Performance during Multi-Session Self-Regulated Learning in the Hypermedia Environment

**DOI:** 10.3390/jintelligence11070131

**Published:** 2023-07-04

**Authors:** Yurou Wang, Haobo Zhang, Jue Wang, Xiaofeng Ma

**Affiliations:** 1 School of Psychology, Northwest Normal University, Lanzhou 730070, China; 2 Key Laboratory of Behavioral and Mental Health, Lanzhou 730070, China; 3 Sleep and NeuroImaging Center, Faculty of Psychology, Southwest University, Chongqing 400715, China; 4 Key Laboratory of Cognition and Personality of Ministry of Education, Southwest University, Chongqing 400715, China

**Keywords:** prompts, feedback, self-regulated learning, hypermedia

## Abstract

The hypermedia environment is among the most prevalent contemporary self-regulated learning (SRL) environments; however, methods for improving the effectiveness of students’ multi-session SRL in such environments remain under discussion. In this study, two experiments were conducted to explore whether and how prompts and feedback benefit performance during multi-session SRL in a hypermedia learning environment. A total of 76 senior students participated in Experiment 1, which used a mixed 2 (prompting condition: prompt, no prompt) × 2 (feedback condition: feedback, no feedback) × 2 (learning session: Session 1 and Session 2) design to explore the effects of prompting and feedback on the multi-session learning process in a hypermedia environment. The results indicated that, in learning Session 1, performance in the prompt condition was significantly better than in the unprompted condition, with or without feedback; in learning Session 2, participants in the prompt condition with feedback performed significantly better than those in the other three conditions. Students in the group with a prompt and feedback had the most accurate meta-comprehension absolute accuracy in both learning sessions. Experiment 2 recruited 94 secondary school students to further explore whether the combination of prompts and different types of feedback led to different learning outcomes according to the division of feedback timing. A mixed 2 (prompt condition: prompt, no prompt) × 3 (feedback condition: delayed feedback, immediate feedback, no feedback) × 2 (learning session: Session 1 and Session 2) design was used. The results indicated that, in learning Session 1, the prompt condition outperformed the unprompted condition with or without feedback; in learning Session 2, students with prompted delayed feedback outperformed the other five conditions. We also found that although there was no significant difference in meta-comprehension monitoring accuracy between delayed and immediate feedback, both groups performed significantly better than those in the no feedback condition. These results suggest that the combination of prompts and feedback in hypermedia environments facilitates student performance better than prompts or feedback alone; this improvement may be related to the correction of poor internal student feedback.

## 1. Introduction

For centuries, improving students’ learning outcomes has been a major focus of educational psychologists. Among various strategies to improve learning outcomes, self-regulated learning (SRL) has attracted widespread attention. Self-regulation refers to planned, cyclic, and self-initiated thoughts, feelings, and actions that individuals engage in to achieve personal goals (Zimmerman 2000). High correlations between self-regulation and learning success have been found in various traditional learning contexts (Bannert et al. 2015; Dignath and Büttner 2008). With the development of science and technology, increasing numbers of researchers have turned their attention in recent years to a new learning medium—hypermedia environments.

The hypermedia environment is a special form of computer-based learning that provides learners with greater control (Scheiter and Gerjets 2007). Hypermedia environments thus require learners to be more active and take the initiative in self-regulation (Scheiter and Gerjets 2007; Shapiro and Niederhauser 2004). The characteristics of hypermedia environments pose a great challenge to students’ learning success in such environments. Whether students are able to self-regulate well largely determines the success of their learning in hypermedia environments (Moos and Azevedo 2008).

Previous research has found that prompts can help students better engage in SRL in hypermedia environments, but the beneficial effects of prompts on SRL seem to be observed only in learning contexts with a single learning phase (short-term effects) (e.g., Azevedo et al. 2011; Bannert and Mengelkamp 2013). Whether prompts lead to good learning outcomes in multiple similar learning situations is still under debate (long-term effects). Some studies have found that prompts can improve learning performance on topics from the domain of educational psychology after three weeks (Bannert et al. 2015; Christoph and Maria 2019), but these results have been challenged by other studies (Breitwieser et al. 2022; Engelmann et al. 2021). For example, Engelmann et al. (2021) did not observe a long-term effect for meta-cognitive prompts in a second learning session after three weeks. According to Breitwieser et al. (2022), the beneficial effects of prompts on learning success are highly variable and may benefit from repetition. However, a recent study using repeated prompts (prompting in both learning sessions) in a hypermedia environment to investigate long-term effects still did not find that meta-cognitive prompts had any significant long-term effects (five days between the two learning sessions). Specifically, Müller and Tina (2018) investigated the effects of cognitive and meta-cognitive prompts on learning outcomes and self-efficacy in two learning phases (five days between the two learning sessions) in a hypermedia environment. Their results showed that although students in the prompt group had significantly higher self-efficacy than those in the unprompted group after the end of the second learning session, there was no significant difference in learning performance.

Previous research has suggested that prompts guide students to engage in meta-cognitive monitoring to generate internal feedback for students to determine whether they need to adjust their knowledge, beliefs, goals, and strategies (Butler and Winne 1995), which plays an important role in SRL. Unfortunately, in multi-session learning—which consists of at least two similar learning sessions with a time lag, in which the learning environment is the same, but the learning materials are different—students do not always have appropriate internal feedback, but may instead produce maladaptive internal feedback. For example, students may have a poorly calibrated self-evaluation (self-assessment, mastery levels of concepts) and may overestimate their learning outcomes, which leads them to stop studying before they have actually mastered the task (Chou and Zou 2020). The primary reason for maladaptive internal feedback is the inaccuracy of students’ meta-cognitive monitoring.

Prompts are a special form of memory and performance aid and are considered strategy activators (Berthold et al. 2007). Prompts usually appear in the form of questions or direct cues to remind learners when and how to engage in productive processing (Thillmann et al. 2009). Although prompts can tell learners when it is appropriate to employ learning strategies, they may only improve learners’ skills in SRL, which does not necessarily mean that students can accurately monitor, control, and regulate their SRL skills (Lee et al. 2010). We thus have a more plausible explanation for the results of the study by Müller and Tina (2018) (i.e., students’ self-efficacy increased at the end of the second session, but learning outcomes did not differ), which may have been due to students’ inaccurate monitoring and their overestimation of learning outcomes, which led them to stop trying in the second learning session.

Providing external feedback may complement the shortcomings of prompts and aid students in engaging in better meta-cognitive monitoring. External feedback is intended to promote student supervision by conveying information about their learning (i.e., observable performance results) and offering opportunities to correct internal feedback, thus reducing the discrepancy between their goals and current performance (Nicol and Macfarlane-Dick 2006). Feedback also serves as a medium for learners to become aware of the cognitive strategies they employ and the efficacy of those strategies, which can prompt them to assess the suitability of the strategies chosen so they can be continued or changed (Narciss 2008). Feedback is a key component in enhancing the acquisition of skills and knowledge (Moreno 2004), and it plays a crucial role in students’ self-regulation. Feedback can be categorized into three types related to knowledge of results (KR), knowledge of the correct response (KCR), and elaborate feedback (EF). Current research has shown that elaborate feedback has the most favorable effect, at least in computer environments (Azevedo and Bernard 1995; Jaehnig and Miller 2007). The learning effect in relation to different timing for feedback is also distinct; research has found that delayed feedback is more effective than immediate feedback in higher-order learning environments (Van der Kleij et al. 2015).

In general, existing research has found that single prompts do not have an ideal beneficial effect on learning outcomes across multiple hypermedia learning sessions. SRL is a continuous learning process in which students reflect on their behavior after the first exam and look forward to better learning performance when encountering similar learning tasks again. It is thus essential to investigate how to help students achieve better learning outcomes in multi-session learning in hypermedia environments.

In this study, we designed and conducted two experiments. To test the hypothesis that the combination of prompts and feedback in multiple learning sessions within hypermedia environments can improve students’ learning outcomes, we conducted Experiment 1. Then, to further distinguish whether the potential effects of different timing for feedback (i.e., delayed feedback may provide better results than immediate feedback), we conducted Experiment 2.

## 2. Experiment 1: The Impact of Prompts and Feedback within Hypermedia Environments on the Performance of Self-Regulated Learning

### 2.1. Experimental Purpose

The experiment was conducted to investigate the effects of prompts and feedback on multi-session learning performance during a two-session learning process in hypermedia environments. Learning performance includes different levels according to Bloom’s taxonomy, from low to high: recall, comprehension, and transfer.

### 2.2. Experimental Method

#### 2.2.1. Participants

The minimum required sample size was 68, as determined by G*power (the F test was selected as “ANOVA: Repeated measures, within-between interaction” and parameters: effect size (Cohen’s d) = 0.3, α = 0.05, power = 0.8). The effect sizes were determined based on two previous meta-analyses where they found effect sizes of 0.32 (Zheng 2016) and 0.49 (Van der Kleij et al. 2015) between prompting and feedback and self-regulated learning performance, respectively, so we conservatively chose 0.3 as the effect size. Considering the potential subject drop-out problem, we planned to recruit 80 high school students as participants. The subjects were randomly assigned to four groups: prompts and feedback, prompts only, feedback only, and control. Due to personal reasons, some participants were unable to complete all experimental tasks and were excluded from the final statistical analysis. The numbers of participants included in the statistical analysis were as follows: 22 participants in the prompts and feedback group and 18 participants in each of the other groups. Detailed demographic information is presented in Table 1.

#### 2.2.2. Experiment Design

The experiment employed a 2 (prompt: with or without prompts) × 2 (feedback: with or without feedback) × 2 (learning session: Session 1 or Session 2) mixed experimental design. The prompting and feedback conditions were between-participants variables and the learning session was a within-participants variable, with learning performance as the dependent variable.

#### 2.2.3. Learning Materials and Environments

**Figure 1:**
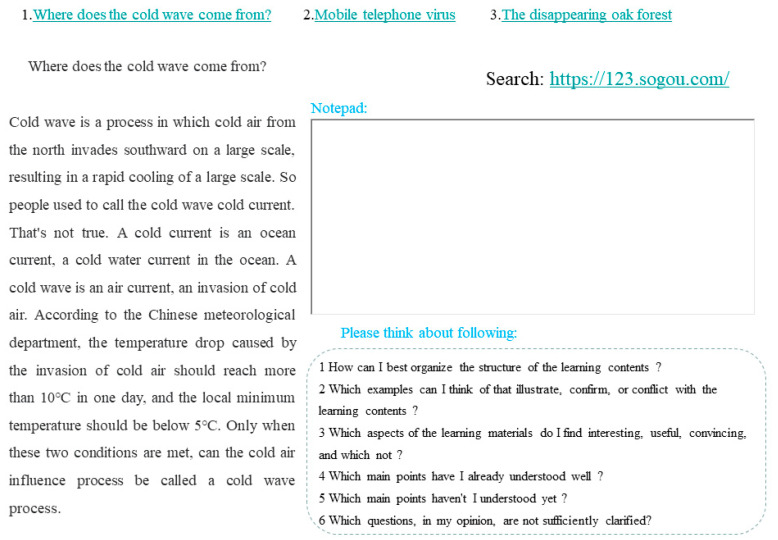
Screenshot of the hypermedia learning environment with an example prompt. Note: In this example (which is translated from Chinese for this paper), the learning content was arranged non-linearly. The learning interface includes a notebook function and a search function, while the bottom right part of the learning interface will always present prompts.

The learning materials consisted of 6 explanatory articles; the first three articles (1. Where does the cold wave come from; 2. Mobile telephone virus; 3. The disappearing oak forest) were learned in the first session, while the last three articles (1. Bone cement; 2. New media; 3. Plants have their joys and sorrows too) were learned in the second session. Hyperlinks connected the pages in a non-linear way, which means subjects can jump to the corresponding study pages by clicking on the hyperlink of the article, without having to follow the sequence (e.g., article 1 must be studied first, then article 2 and finally 3). The learning environment also includes a search function (allows subjects to search the Internet when they want to expand on their learning) and notepad, as shown in Figure 1.

All six explanatory articles were selected from authoritative teaching materials recommended by high school Chinese language teachers for students at the reading level of the subjects participating in the study, with necessary modifications made by the researchers to the original text and titles.

In order to ensure the reliability and validity of the materials, high school Chinese language teachers were first invited to screen the reading materials used in the experiment. Eight explanatory articles that were suitable for the reading comprehension level of first-year high school students were selected and were distributed to forty non-experimental students for them to rate the difficulty level of each article from 1 (very easy) to 5 (very difficult). After screening out the articles that were either too easy or too difficult, six explanatory articles were ultimately selected as the experimental materials.

In addition, five teachers were invited to rate the content validity of the test questions (a total of nineteen questions) for the six reading materials. Then, the Kendall’s coefficient of concordance was used to assess the consistency of the five raters, and the results showed that the rating of the test questions was consistent, W = 0.812, df = 18, *p* < 0.05. The results indicated that the test questions could be used as measurement indicators to evaluate the learning performance in this study. Furthermore, to evaluate the prior knowledge, we randomly selected 7 questions from 19 to assess prior knowledge (3 questions for Session 1 and 4 questions for Session 2) while the remaining 12 questions were used for the final test (6 questions for each session) to evaluate the learning effects in terms of recall, comprehension, and transfer.

#### 2.2.4. Prompt Setting

The prompting condition consisted of a combination of three cognitive prompts and three meta-cognitive prompts, which were shown to be particularly successful in activating self-regulatory activities (Berthold et al. 2007). One cognitive prompt was to prompt organization of content (“How can I best organize the structure of learning content?”), and two clues were provided (for example, “What examples can I come up with to explain, confirm, or conflict with learning content?”). Three meta-cognitive prompts were provided for clue monitoring (for example, “Do I understand the key points well enough?”). The prompts were linked to specific pages in the learning environment and displayed in frames directly below the title. Additionally, it should be noted that the same prompts were used on each page for all 6 articles learned, and a prompt was always present in the learning interface that did not require any additional action by the student.

#### 2.2.5. Feedback Setting

According to the feedback type, the feedback condition received detailed feedback, for example: it was mentioned in the text that hyperlinks do not have a standardized order and contain countless combinations of reading paths. The establishment of a specific reading path depends entirely on the reader’s specific reading intentions and aesthetic preferences. Therefore, the option that a specific reading order in the text can immerse readers better in the text content is a distortion of the original text. Therefore, option C is incorrect, and options A, B, D are the content of the original text. In addition, to ensure that participants actually processed the provided feedback, we asked them to read the feedback for at least 5 min. Feedback was provided whether or not the subject’s answer was correct in feedback condition. In contrast, no feedback was provided to participants during testing in the no feedback condition.

#### 2.2.6. Learner Characteristics Assessment

To control for the participants’ learning characteristics and to avoid any impacts on the experimental results caused by their existing learning traits, the study measured the participants’ learning strategies, motivation, and prior knowledge using three approaches.

*Learning Strategy*: The Learning Strategies Scale, designed by Qin of the Institute of Developmental Psychology at Beijing Normal University, was used to measure participants’ learning strategies. This scale divided learning strategies into four dimensions: meta-cognitive strategies (referring to monitoring, regulation, planning measures, e.g., item: Making a good study plan), cognitive strategies (referring to specific learning methods, e.g., item: Following the teacher closely in class and think positively), motivational strategies (referring to attitude and motivation, e.g., item: Taking exams seriously), and social strategies (referring to help from others, extensive social learning, e.g., item: Asking teachers and classmates if you don’t understand something). This scale was designed for high school students with clear dimension divisions and has high reliability and validity. In Experiments 1 and 2, the Cronbach alpha was 0.76 and 0.75, respectively. It has been widely used in related studies of learning strategies and includes 52 items, using a 5-point scoring system ranging from “always” (5 points) to “never” (1 point). Higher scores indicate better levels of learning strategy development.

*Learning Motivation*: The study’s assessment of learning motivation used the Learning Motivation Scale. This scale was first developed by Biggs (1987) and later revised by Li et al. (1997), with good reliability and validity. In Experiments 1 and 2, the Cronbach alpha was 0.74 and 0.81, respectively. The scale includes three dimensions: surface-level learning motivation (refers to the desire to cope with examinations and motivation to study to pass exams, e.g., item: My goal is to pass the exam with the least amount of effort), deep-level learning motivation (refers to the motivation to learn in order to understand and master the content with an intrinsic interest in it, e.g., item: I often find learning about knowledge as exciting as watching a novel or a film movie), and achievement-oriented learning motivation (refers to the motivation to learn in order to achieve high marks and receive praise, e.g., item: I find that studying often gives me a sense of personal satisfaction), with a 5-point scoring system ranging from “always” (5 points) to “never” (1 point).

*Prior knowledge*: Prior knowledge was assessed before each learning session to ensure that there were no prior differences in the students’ mastery of the upcoming content. For Session 1, prior knowledge was evaluated through 2 multiple-choice questions (3 points each) and 1 open-ended question (4 points), while for Session 2, prior knowledge was evaluated via 3 multiple-choice questions (4 points each) and 1 open-ended question (4 points). All questions were evaluated by the teacher to ensure that prior knowledge could be effectively measured, as detailed in Section 2.2.3 on Learning Materials and Environment. Total scores were calculated for each assessment and used for statistical analysis.

#### 2.2.7. Learning Performance Assessment

Measurements were conducted through two paper-based tests, which were administered after each learning session. Both tests evaluated learning performance from three levels (recall, comprehension, transfer) based on Bloom’s taxonomy (Bloom 1956). All open-ended questions were scored by an experienced secondary school teacher, who gave scores on a point-by-point basis with reference to standard answers.

The performance evaluation after the first study session included six questions, which were multiple-choice or open-ended questions about the first three hypermedia materials. Recall was measured through two open-ended questions about basic concept definitions (e.g., “What are the three ways in which mobile phone viruses attack and cause harm?”). Comprehension was measured by two multiple-choice questions (e.g., “Which of the following is not correct regarding the analysis of the ‘Cabir’ mobile phone virus?”). Transfer was measured by one multiple-choice question and one open-ended question, in which learners had to apply what they learned to new situations (e.g., “What are the main functions of anthocyanins in blueberries?”). The maximum score is 15 points: recall = 4 points, comprehension = 4 points, and transfer = 7 points.

The performance evaluation after the second study session included six questions, which were multiple-choice or open-ended questions about the last three hypermedia materials. Recall was measured through two open-ended questions about basic concept definitions (e.g., “Why do we say that all plants are ‘lustful’?”). Comprehension was measured by two multiple-choice questions (e.g., “Which of the following statements about hypermedia reading is incorrect?”). Transfer was measured by two questions in which learners had to apply what they learned to new situations. The maximum score is 15 points: recall = 4 points, comprehension = 4 points, and transfer = 7 points.

#### 2.2.8. Absolute Accuracy of Meta-Cognitive Monitoring

The absolute accuracy in this study was calculated using the absolute accuracy in predicting the total score, that is, the predicted value of the score minus the actual total value of the score yields a predicted actual (PA). A positive PA indicates overconfidence, whereas a negative one indicates underconfidence. The absolute value represents the degree of deviation between the judgment and the score (Maki et al. 2005).

#### 2.2.9. Experiment Procedure

**Figure 2:**
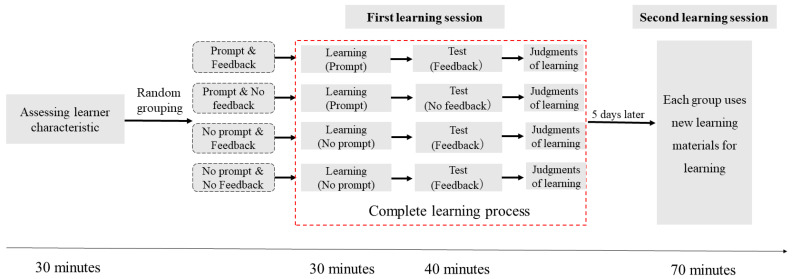
Schematic diagram of the Experiment 1 process.

The experimental procedure is shown in Figure 2. Firstly, the participants’ learning characteristics were obtained through an online questionnaire survey. Learning strategies and motivation were measured with the tools mentioned above. The online survey took approximately 30 min to complete.

After evaluating the participants’ learning characteristics, learning took place in a face-to-face course. Participants were seated at desks equipped with laptops. Participants were informed of the experiment, completed the prior knowledge test, and watched an introductory video about the learning environment, which included information about what functions are available in the hypermedia learning environment (e.g., the content, navigation, search function, and notepad), how each feature is used, what to expect during the learning process (e.g., no communication with each other and no access to other tools), and how long they have available for learning. Next, participants learned for 30 min in the hypermedia environment and, at the end of 30 min, the learning process was ended by a researcher. During the learning session, the intervention group received six different self-regulation prompts while the control group learned without any prompts. After the learning session finished, performance tests were administered (not exceeding a maximum of 40 min and subjects could confirm the time spent by looking at the clock hanging at the front of the classroom), and detailed feedback was provided to the feedback group. Finally, learners evaluated their own performance. The experimental task lasted for approximately 70 min.

The second learning session took place five days after the first one. In this session, students had to learn another three sections in the same learning environment as the first session. The procedure was the same as the first learning session, starting with an evaluation of prior knowledge. Next, the participants received instructions. Then, they started the 30 min of learning. After the learning session finished, performance tests were administered, and detailed feedback was provided to the feedback group. Finally, learners evaluated their own performance (range: 0–15, based on overall test score) after studying the feedback. The task lasted approximately 70 min.

### 2.3. Results

#### 2.3.1. Comparison of Controlled Variables between Groups

A one-way ANOVA was conducted on the gender, age, learning strategies, motivation, and prior knowledge of four groups of participants before the start of two learning sessions. Results indicated no significant differences among the groups in the aforementioned control variables. Details of the results from the ANOVA are presented in Table 1.

#### 2.3.2. Three-Factor ANOVA of Different Learning Metrics

Three-factor ANOVA was conducted on the recall, comprehension, and transfer, with prompting condition, feedback condition, and learning session analyzed as factors. Descriptive statistics for each metric are presented in Table 2, while the results of the ANOVA are summarized in Table 3. For recall and comprehension, no significant main or interaction effects were detected. However, for transfer, a significant interaction was observed between the prompting condition, feedback condition, and learning session. Therefore, the subsequent analysis will explore the impact of prompting and feedback conditions on transfer in different learning sessions.

**Figure 3:**
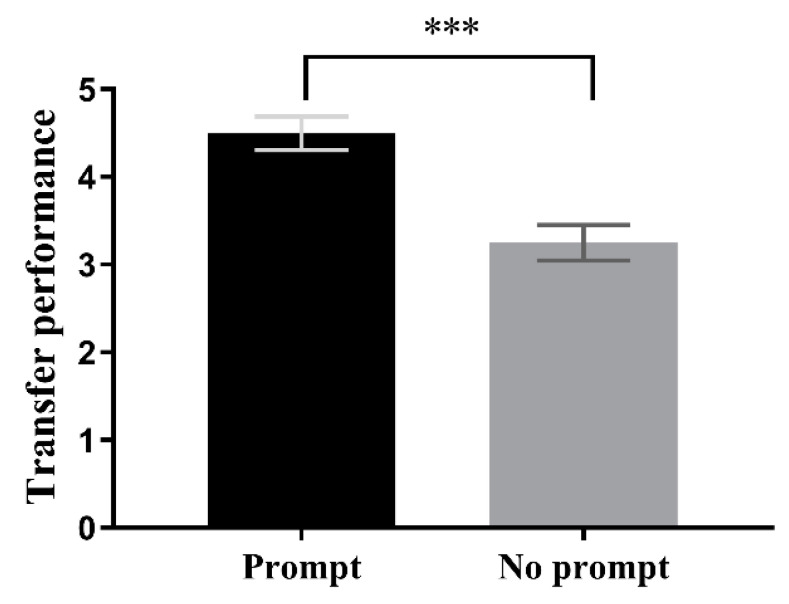
Transfer performance in learning Session 1 is better with than that without the prompt condition, whether feedback is provided or not. *** *p* < 0.001.

A two-factor ANOVA was conducted with prompting condition and feedback condition as independent variables and transfer metric from learning Session 1 as the dependent variable. Results indicated that the prompting condition had a significant main effect, F(1,72) = 20.117, *p* < 0.001, *η*^2^ = 0.22. However, the feedback condition did not have a significant main effect, F(1,72) = 2.217, *p* = 0.141, *η*^2^ = 0.03, nor was there a significant interaction effect (Fs < 1). Follow-up analysis of the main effect of prompting condition showed that performance on the transfer test for participants in the prompting condition (M = 4.50, SD = 0.19) was significantly better than that of participants in the non-prompting condition (M = 3.25, SD = 0.20), F(1,72) = 20.18, *p* < 0.001, *η*^2^ = 0.22, 95CI = [0.69, 1.80], as shown in Figure 3.

Using transfer in learning Session 2 as the dependent variable and prompting and feedback conditions as the independent variables, a two-factor ANOVA was conducted. The results of the ANOVA showed a significant main effect for the prompting condition, F(1,72) = 23.655, *p* < 0.001, *η*^2^ = 0.25, and a significant main effect for the feedback condition, F(1,72) = 13.786, *p* < 0.001, *η*^2^ = 0.16. Additionally, the interaction effect between the two conditions was also significant, F(1,72) = 5.546, *p* < 0.05, *η*^2^ = 0.07. Simple effects analyses were conducted in both directions for this interaction effect.

**Figure 4:**
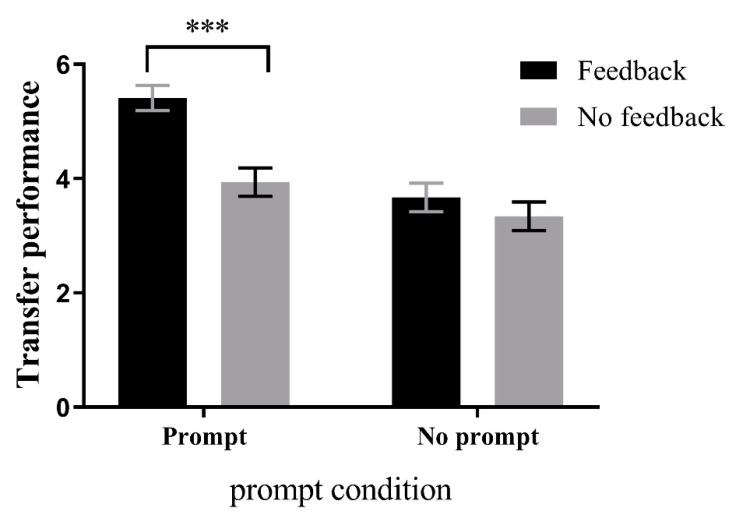
Simple effects analysis in the direction of the prompt condition in learning Session 2 transfer performance. Giving feedback will result in better transfer performance than no feedback under prompting condition. However, there was no significant difference whether feedback was given or not under no prompting condition. *** *p* < 0.001.

*Prompting condition*: Under the prompting condition, the feedback group (M = 5.41, SD = 0.22) performed significantly better than the no feedback group (M = 3.94, SD = 0.25), F(1,72) = 19.33, *p* < 0.001, *η*^2^ = 0.21, 95CI = [0.80, 2.13]. However, under the no prompting condition, there was no significant difference between the feedback group (M = 3.67, SD = 0.25) and the no feedback group (M = 3.34, SD = 0.25), Fs < 1, as shown in Figure 4.

*Feedback condition*: Under the feedback condition, the prompting group performed significantly better than the no prompt group, F(1,72) = 27.36, *p* < 0.001, *η*^2^ = 0.28, 95CI = [1.08, 2.41]. However, under the no feedback condition, there was no significant difference between the prompt group and the no prompt group, F(1,72) = 3.00, *p* = 0.09, *η*^2^ = 0.04, 95CI = [−0.09, 1.30].

#### 2.3.3. Three-Factor ANOVA of Predicted Actual

A three-factor ANOVA with prompting condition, feedback condition, and learning phase was conducted on PA. Descriptive statistics are shown in Table 2 and ANOVA results are presented in Table 3. The results indicated a significant main effect of feedback condition and a significant interaction effect between the prompting condition and feedback condition, but no significant three-way interaction effect (Table 3). Therefore, the subsequent analysis was conducted on significant interaction effects to explore the influence of the prompting and feedback conditions on PA.

**Figure 5:**
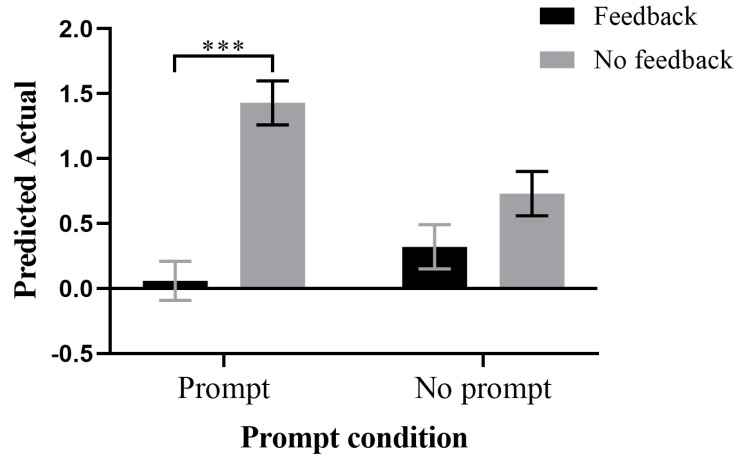
Interaction effects of prompt and feedback conditions on predicted actual (the predicted value of the score minus the actual total value of the score), with a simple effects analysis in the direction of the prompt condition. *** *p* < 0.001.

Simple effects analyses were conducted from two directions. Firstly, under the prompting condition, the PA of the feedback group (M = 0.06, SD = 0.15) was significantly lower than that of the no feedback group (M = 1.43, SD = 0.17), F(1,72) = 36.06, *p* < 0.001, *η*^2^ = 0.33. However, under the no prompting condition, there was no significant difference between the feedback group (M = 0.32, SD = 0.17) and the no feedback group (M = 0.73, SD = 0.17) in terms of the PA, F(1,72) = 2.98, *p* = 0.089, *η*^2^ = 0.04, as shown in Figure 5.

*Feedback condition*: Under the no feedback condition, the PA of the prompt group was significantly higher than that of the no prompt group, F(1,72) = 8.445, *p* < 0.01, *η*^2^ = 0.11. However, under the feedback condition, there was no significant difference between the prompt group and the no prompt group in terms of the PA, F(1,72) = 1.318, *p* = 0.255, *η*^2^ = 0.02.

### 2.4. Discussion

In Experiment 1, the effects of self-regulated prompting and feedback on learning performance in two hypermedia learning sessions were explored. Based on previous research, we hypothesized that in Session 1, the prompting condition would be superior to the no prompting condition, and feedback condition would have no effect on learning performance. Meanwhile, in Session 2, the combination of prompts and feedback leads to better learning performance.

As expected, there were differences in learning performance among groups in terms of transfer in Session 1; however, there were no significant differences in the recall and comprehension among groups. The result is consistent with previous research (Müller and Tina 2018). In Session 2, there was a significant interaction effect between prompting and feedback conditions. The group with both prompting and feedback outperformed the other groups, while the group with neither prompting nor feedback performed the worst. The results showed that providing both prompting and feedback led to better transfer performance in the context of self-regulated hypermedia learning environments.

Furthermore, we found that students’ meta-comprehension absolute accuracy was significantly higher when provided with feedback than under the no feedback condition. This suggests that external feedback may correct students’ inaccurate internal feedback and improve their meta-comprehension absolute accuracy, which may be a potential reason for the significant improvement in transfer performance.

Overall, Experiment 1 validated our hypothesis but had some limitations. We did not specifically examine different feedback timing. Feedback can be divided into immediate feedback and delayed feedback based on timing, and different feedback timing has been found to have different effects on learning performance in previous research. Therefore, in Experiment 2, we further explored which combination of feedback type and prompts can lead to better learning outcomes based on different feedback timings.

## 3. Experiment 2: The Impact of Prompts and Different Types of Feedback on Self-Regulated Learning Outcomes in a Hypermedia Environment

### 3.1. Experiment Purpose

Based on Experiment 1, we further explored whether there are significant differences in learning performance when prompts are combined with feedback at different times.

### 3.2. Experiment Method

#### 3.2.1. Participants

The minimum required sample size was 78, as determined by G*power (the F test was selected as “ANOVA: Repeated measures, within-between interaction” and parameters: effect size (Cohen’s d) = 0.3, α = 0.05, power = 0.8). The effect sizes were determined based on the same rule used in Experiment 1. Considering the potential subject drop-out problem, we planned to recruit 108 high school students (none of them was involved in Experiment 1) as participants, who would be randomly distributed into six groups (prompted with no feedback, immediate feedback, and delayed feedback; not prompted with no feedback, immediate feedback, and delayed feedback). Due to various reasons, 14 participants were excluded from the final statistical analysis (5 subjects were not in school at the time of the second session, 3 subjects did not wish to continue to participate, and 6 subjects did not complete all test items during the testing phase), and the numbers of participants included in each group for statistical analysis were as follows: 15 participants in the prompt with delayed feedback group and prompted with no feedback group and 16 participants in each of the other groups, as shown in Table 4 for detailed demographic information.

#### 3.2.2. Experiment Design

The experiment utilized a 2 (prompting condition: prompting, not prompting) × 3 (feedback condition: delayed feedback, immediate feedback, no feedback) × 2 (learning session: Session 1, Session 2) mixed experimental design. The prompting condition and feedback condition were between-participants variables, the learning session was a within-participants variable, and learning performance was the dependent variable.

#### 3.2.3. Learning Materials and Environments

The learning materials and environments were the same as in Experiment 1.

#### 3.2.4. Prompt Setting

The prompts were the same as in Experiment 1.

#### 3.2.5. Feedback Setting

The feedback content was consistent with Experiment 1, with the difference that the timing of feedback was subdivided into two types: immediate feedback (similar to Experiment 1) and delayed feedback (provided after one day of testing) in this experiment.

#### 3.2.6. Learner Characteristics Assessment

The learner characteristics assessment was the same as in Experiment 1.

#### 3.2.7. Learning Performance Assessment

The learning performance assessment was the same as in Experiment 1.

#### 3.2.8. Absolute Accuracy of Meta-Cognitive Monitoring

The absolute accuracy of meta-cognitive monitoring was the same as in Experiment 1.

#### 3.2.9. Experimental Procedure

**Figure 6:**
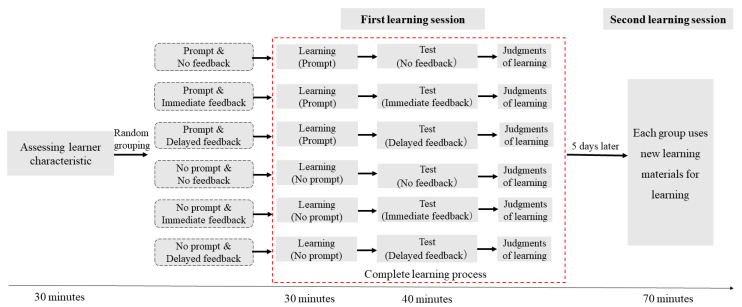
Schematic diagram of the Experiment 2 process.

The experimental procedure is illustrated in Figure 6. Firstly, learner characteristics were obtained through an online questionnaire survey. Learning strategies and motivation were measured with the tools mentioned above. Completing the online questionnaire took approximately 30 min.

After assessing the learner characteristics of the participants, the learning took place in a face-to-face course, with participants seated at desks equipped with laptops. Participants were informed about the experiment, completed the previous knowledge test, and watched an introductory video about the learning environment that included information about the content, navigation, search function, and notepad. Then, participants received written instructions to learn as much as possible from the first four sections. Next, participants learned for 30 min in the hypermedia environment, with the intervention group receiving six different self-regulation prompts while the control group learned without any prompts. After the learning session finished, performance tests were administered, and detailed feedback was provided to the feedback group. Finally, learners evaluated their own performance. The experimental task lasted for approximately 70 min.

The second learning session took place five days after the first one. In this session, students had to learn four additional sections in the same learning environment as the first session. The procedure was same as the first learning session, starting with an evaluation of prior knowledge. Next, the participants received instructions. Then, they started the 30 min of learning. After the learning session finished, performance tests were administered, and detailed feedback was provided to the feedback group. Finally, learners evaluated their own performance. The task lasted approximately 70 min.

### 3.3. Results

#### 3.3.1. Comparison of Controlled Variables between Groups

A one-way ANOVA was conducted on the variables of sex, age, learning strategies, learning motivation, and prior knowledge before the start of the two learning sessions for the six groups of participants, and the results showed that there were no significant differences among the groups in the aforementioned control variables. The results are presented in Table 4.

#### 3.3.2. Three-Factor ANOVA of Different Learning Metrics

A three-way ANOVA of prompting condition, feedback condition, and learning sessions was performed on the recall, comprehension, and transfer. The descriptive statistics of each measure are presented in Table 5, and the ANOVA results are displayed in Table 6. Results showed that only the main effect of learning sessions was significant for the recall; no significant main or interaction effects were found for the comprehension; multiple significant effects were found for the transfer, and the interaction of condition × feedback condition × learning sessions was also significant. Therefore, the subsequent analysis will focus on the effects of prompting condition and feedback condition on the transfer measure in different learning sessions.

Regarding the recall, a main effect of learning session was found. The score for learning Session 2 (M = 2.16, SD = 0.09) was significantly higher than that of learning Session 1 (M = 2.01, SD = 0.10), F(1, 88) = 8.26, *p* < 0.01, *η*^2^ = 0.09.

**Figure 7:**
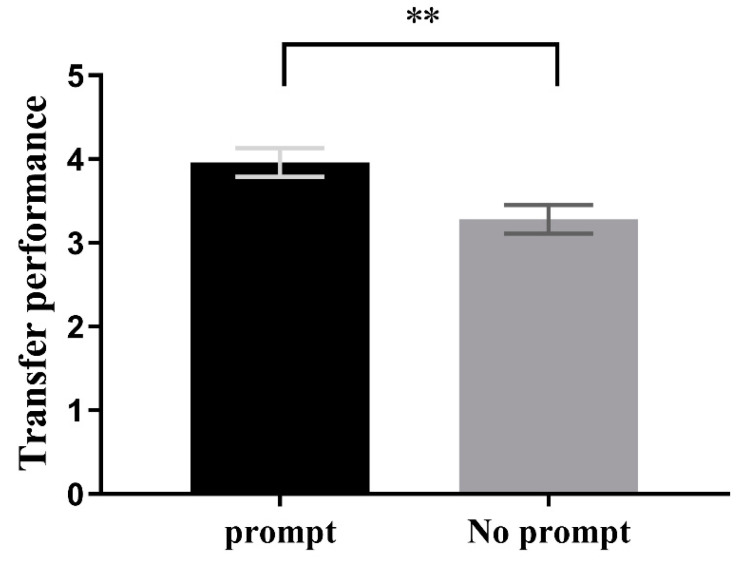
Transfer performance on learning Session 1 is better with than without the prompt condition. ** *p* < 0.01.

A two-way ANOVA was conducted with prompting condition and feedback condition as independent variables and the transfer of learning Session 1 as the dependent variable. The results showed a significant main effect of prompting condition, F(1, 88) = 8.02, *p* < 0.01, *η*^2^ = 0.08, but no significant main effect of feedback condition, Fs < 1, and no significant interaction effect between the two, Fs < 1. Further analysis showed that in the prompting condition, the performance of participants in the transfer test (M = 3.96, SD = 0.17) was significantly better than that in the no prompting condition (M = 3.28, SD = 0.17), F(1, 88) = 8.02, *p* < 0.01, *η*^2^ = 0.08, 95%CI = [0.20, 1.16], as shown in Figure 7.

The transfer of learning Session 2 was used as the dependent variable and the prompting condition and feedback condition were used as independent variables in a two-way ANOVA. The results showed a significant main effect of prompting condition, F(1, 88) = 45.637, *p* < 0.001, *η*^2^ = 0.34, and a significant main effect of feedback condition, F(1, 88) = 24.482, *p* < 0.001, *η*^2^ = 0.36. Additionally, the interaction effect between the two was significant: F(1, 88) = 5.230, *p* < 0.01, *η*^2^ = 0.11. Therefore, simple effects analyses were conducted from two directions to explore the interaction effect.

**Figure 8:**
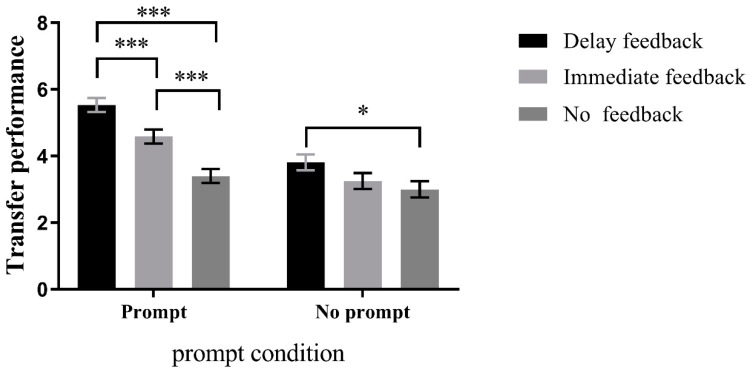
Simple effects analysis in the direction of the prompt condition in learning Session 2 transfer performance. *** *p* < 0.001, * *p* < 0.05.

*Prompting condition*: There was a significant difference among the delayed feedback, immediate feedback, and no feedback groups in the prompting condition, F(1, 88) = 24.996, *p* < 0.001, *η*^2^ = 0.36. Post hoc comparisons revealed that the delayed feedback group (M = 5.53, SD = 0.21) performed significantly better than the no feedback group (M = 3.40, SD = 0.21), *p* < 0.001, 95%CI = [1.40, 2.87], and the immediate feedback group (M = 4.59, SD = 0.21) performed significantly better than the no feedback group, *p* < 0.001, 95%CI = [0.47, 1.92]. In addition, the delayed feedback group performed significantly better than the immediate feedback group, *p* < 0.01, 95%CI = [0.21, 1.67]. In the no prompting condition, there was also a significant difference among the three groups, F(1, 88) = 4.038, *p* < 0.05, *η*^2^ = 0.08. Post hoc comparisons showed that the delayed feedback group (M = 3.81, SD = 0.24) performed significantly better than the no feedback group (M = 3.00, SD = 0.24), *p* < 0.05, 95%CI = [0.10, 1.53]. However, there was no significant difference between the delayed feedback group and the immediate feedback group (M = 3.25, SD = 0.24), *p* = 0.174, 95%CI = [−0.15, 1.28], and there was no significant difference between the immediate feedback group and the no feedback group, *p* = 1.000, 95%CI = [−0.47, 0.97]. These results are presented in Figure 8.

**Figure 9:**
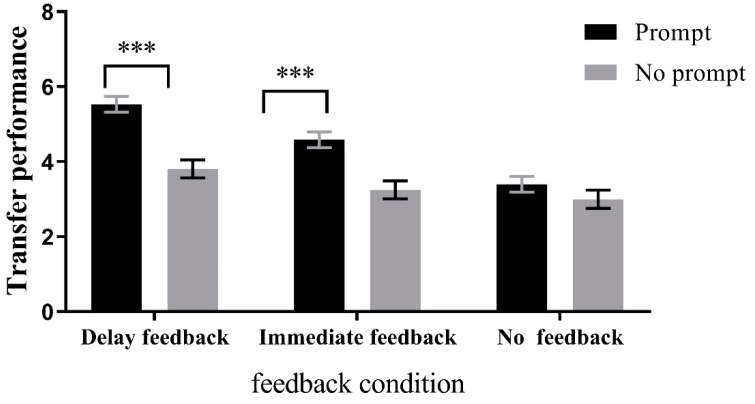
Simple effects analysis in the direction of feedback conditions in learning Session 2 transfer performance. *** *p* < 0.001.

*Feedback condition*: For the delayed feedback condition, participants in the prompting group (M = 5.53, SD = 0.21) significantly outperformed those in the non-prompting one (M = 3.81, SD = 0.21), F(1,88) = 33.413, *p* < 0.001, *η*^2^ = 0.28, 95CI = [1.13, 2.31]. For the immediate feedback condition, participants in the prompting group (M = 4.59, SD = 0.21) significantly outperformed those in the non-prompting group (M = 3.25, SD = 0.21), F(1,88) = 21.053, *p* < 0.001, *η*^2^ = 0.19, 95CI = [0.76, 1.93]. However, no significant difference was found between the prompting and non-prompting groups under the no feedback condition, F(1,88) = 1.805, *p* = 0.183, *η*^2^ = 0.02, 95CI = [−0.19, 0.99], as shown in Figure 9.

#### 3.3.3. Three-Factor ANOVA of PA

A three-factor ANOVA was conducted on the PA, with prompting condition, feedback condition, and learning sessions analyzed as factors. Descriptive statistics are presented in Table 5 while the ANOVA results are summarized in Table 6. The ANOVA result showed a significant main effect of feedback condition, but no other significant main or interaction effects were found (Table 6). Therefore, the following analysis will focus on the main effect of feedback condition.

**Figure 10:**
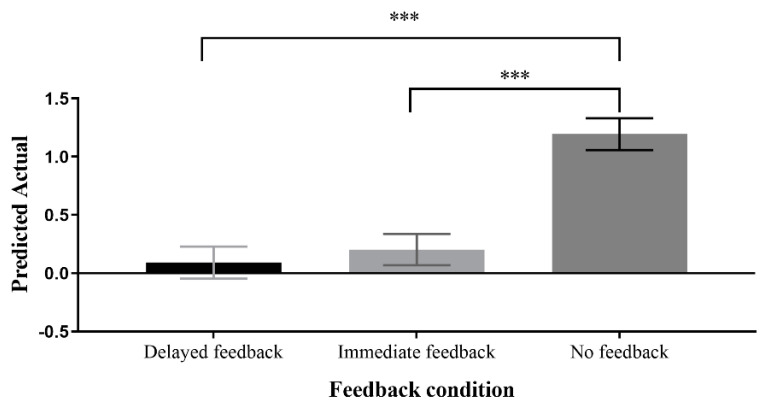
Post hoc analysis of the main effects of feedback conditions on predicted actual (the predicted value of the score minus the actual total value of the score). *** *p* < 0.001.

The main effect of feedback condition was investigated through follow-up analyses. The results showed that the PA was significantly better in the delayed feedback condition (M = 0.09, SD = 0.14) compared to the no feedback condition (M = 1.19, SD = 0.14), *p* < 0.001, 95CI = [−1.57, −0.63]. However, no significant difference was observed between the delayed feedback condition and immediate feedback condition (M = 0.20, SD = 0.13), *p* = 1.00, 95CI = [−0.58, 0.36]. In addition, a significant difference was found between the immediate feedback condition and the no feedback condition, *p* < 0.001, 95CI = [−1.46, −0.52]. These results indicate that for PA, delayed feedback and immediate feedback are comparable and both superior to the no feedback condition, as shown in Figure 10.

### 3.4. Discussion

According to the results of the study, we explored the combination of self-regulated prompts and feedback types (delayed or immediate feedback) that are best suited for students in a self-regulated learning environment such as hypermedia. In this study, we found that the group with self-regulated prompts and delayed feedback had better learning outcomes, compared with the other five groups.

Based on previous research, the main effect of prompting conditions was significant in learning Session 1. Experiment 2 confirmed the conclusions of previous studies and Experiment 1, verifying that prompts can indeed improve students’ learning performance in a single learning environment. In Session 2, the interaction between prompting and feedback conditions was significant, indicating that delayed feedback was the most effective with prompts. Additionally, the absolute accuracy of meta-comprehension was significantly better in the delayed feedback and immediate feedback conditions than in the no feedback condition, which is similar to the results of most previous studies. When feedback is aimed at promoting lower-level learning outcomes, immediate feedback is most effective, but when higher-level learning outcomes are affected, delayed feedback is best. In the hypermedia situation, a complex learning environment, delayed feedback is required.

## 4. General Discussion

Previous research has emphasized the importance of SRL for learning success. Prompts have been identified as one teaching method that can promote SRL. While the use of prompts to promote SRL and improve learning performance has been widely studied, the results of such studies in the hypermedia environment with multiple learning sessions are not very promising. This study was conducted in a hypermedia environment that included two learning sessions. Two experiments were conducted to explore whether self-regulated prompts and feedback could improve students’ absolute meta-comprehension accuracy in an SRL environment such as hypermedia, thereby promoting learning performance during multiple learning sessions.

Experiment 1 recruited high school students as participants and employed a three-factor mixed 2 (prompting condition: with and without prompts) × 2 (feedback condition: with and without feedback) × 2 (learning session: Session 1 and Session 2) design to explore whether the combination of self-regulated prompts and feedback would lead to better learning performance in a hypermedia environment. The results showed that the group with prompts performed the best in Session 1, while the group with both prompts and feedback showed the best performance in Session 2, which was consistent with our hypothesis. Additionally, students in the group with feedback had the best absolute meta-comprehension accuracy.

Experiment 2 was conducted with high school students as participants in a mixed 2 (prompting condition: with and without prompts) × 3 (feedback condition: delayed feedback, immediate feedback, and no feedback) × 2 (learning session: Session 1 and Session 2) design, building on the design of Experiment 1. The experimental environment was the same as in Experiment 1, but we explored which type of feedback, in combination with the prompt, would lead to better learning performance based on different feedback timing. The results showed that, in learning Session 1, the conclusion of Experiment 1 was confirmed; prompts improve students’ learning outcomes in single learning sessions. In learning Session 2, with the prompting condition, the delayed feedback group showed better performance than the immediate or no feedback groups. The absolute meta-comprehension accuracy was also significantly better in both the delayed feedback group and the immediate feedback group than in the no feedback group. However, there was no significant difference between the immediate and delayed feedback groups.

### 4.1. Prompts and Feedback Can Continuously Improve Self-Regulated Learning Performance in Multi-Session Learning

The results of Experiment 1 showed that the only differences in the transfer were found among the groups in learning Session 1. This is consistent with the conclusions of previous studies on prompts, which indicated that prompts support deeper processing and self-regulation activities based on enhanced monitoring (Bannert et al. 2009; Berthold et al. 2007). However, prompts seemed only to stimulate the activation of self-regulation strategies at a more refined processing level. This deeper level of processing—such as thinking about examples or monitoring whether a concept is understood, rather than understanding some important terms in the text better—may lead learners to focus only on information relevant to the transfer, thus promoting transfer-related indicators. Another reason why no significant differences were found for the comprehension indicators may be due to the fact that our test questions were all multiple choice and there were no open-ended questions. This means that although students chose most of the correct options, they would not score well so long as they chose one wrong option. Having open-ended questions may be more suitable for evaluating students’ comprehension indicators, as it would make it possible to rate students based on their specific level of understanding.

In learning Session 2, the interaction between the prompting and feedback conditions was significant. The group with both prompts and feedback had better performance than the other groups, while the group with neither prompts nor feedback performed the worst. This contrasts with the results of the study by Müller and Tina (2018), which investigated the effects of self-regulated prompts on students’ self-efficacy and learning performance in two hypermedia learning sessions. In Session 2, their results showed that prompts did not lead to better learning performance, and there was no significant difference between the prompted and unprompted groups. However, students’ self-efficacy increased, which led to overestimation of their learning performance and cessation of studying before the task ended.

In our study, we therefore provided both prompts and timely feedback to help students understand their learning progress, which improved their performance in Session 2, as expected. We measured students’ meta-comprehension accuracy and learning indicators and found that students in the no feedback group generally had poor learning judgments, no matter the prompt condition. Additionally, students in the feedback group had accurate PA and the best performance. As previously stated, prompts guide students to engage in meta-cognitive monitoring, which generates internal feedback to establish whether adjustments in knowledge, beliefs, goals, or strategy are required (Butler and Winne 1995). Unfortunately, students do not always have appropriate internal feedback during multi-session learning processes; instead, they are more likely to generate maladaptive internal feedback. External feedback provides key information that helps students calibrate the differences between knowledge acquisition and learning goals. This improved calibration reduces uncertainty during the learning task, leading to increased motivation and continued learning until the task is complete. Our study improved students’ inadequate internal feedback by providing external feedback, resulting in improved learning outcomes at the end of learning Session 2.

Generally speaking, for learners in computer-based learning environments, simple prompts may not provide enough guidance, because learners need to monitor, understand, and adjust their own learning process based on their performance, which was supported by Experiment 2. However, different timing for the feedback can also affect different learning outcomes. Experiment 1 only verified that prompts and feedback can lead to good learning performance, without considering the timing of the feedback. Experiment 2 therefore further verified which combination of feedback types and prompts can yield better learning performance based on different timing for feedback.

### 4.2. Prompts and Delayed Feedback Can Consistently Enhance Self-Regulated Learning Performance in Multi-Session Learning

In Experiment 2, we divided the timing of the feedback into immediate and delayed. The results once again confirmed our first hypothesis that the main effect of prompts in learning Session 1 was significant. This again demonstrated that prompts can lead to good learning performance in a single-session learning context. However, SRL is a continuous process of learning and reflection for students, who expect to improve their learning performance when encountering similar learning tasks again after the first exam. Clearly, prompts alone are far from sufficient in multi-session hypermedia environments.

In learning Session 2, we verified our fourth hypothesis that the interaction between the prompting condition and the feedback condition was significant. The group with delayed feedback and prompts showed the best performance, while the group with no prompts and no feedback performed the worst. Hattie and Timperley (2007) suggested that delayed feedback is better for difficult tasks, because such tasks involve greater task processing than simple tasks, and delayed feedback provides the opportunity to perform that processing. Similarly, Shute (2008) argued that immediate feedback is most effective when promoting lower-level learning outcomes, but delayed feedback is preferable when higher-level learning outcomes are threatened. A complex hypermedia learning environment thus requires delayed feedback, because when students are dealing with higher-level tasks, delayed feedback is more effective than immediate feedback (Van der Kleij et al. 2015). Delayed feedback can strengthen students’ psychological representations and create new opportunities for better understanding and more comprehensively articulating learning materials (Butler et al. 2013; Kapp et al. 2015). Delayed feedback also focuses more on long-term knowledge acquisition in a more comprehensive way (Butler et al. 2007; Metcalfe et al. 2009; Shute 2008). As previously mentioned, delayed feedback allows students to engage in new opportunities for relearning and consciously monitoring the entire learning process (Kulhavy and Anderson 1972). The theoretical models in feedback research also emphasize the effectiveness of the delay cycle between these cases and feedback for self-regulation and learning (Kapp et al. 2015; Narciss 2013). For example, Kulhavy and Anderson (1972) emphasized that when students are provided delayed feedback, they are more likely to cognitively exclude incorrect answers from memory, which results in obtaining the correct answers more fluently.

Interestingly, there was no significant difference in the absolute accuracy of meta-comprehension between the delayed and immediate feedback conditions, but both were better than the no feedback group. Although the results did not support the notion that delayed feedback would result in better absolute accuracy in meta-comprehension (tendency but not significant difference), they did indicate that feedback improved learning performance by correcting the absolute meta-comprehension accuracy.

In conclusion, providing students with delayed feedback and prompts in the complex hypermedia learning environment helps students better monitor their learning process, improves the absolute accuracy of their meta-comprehension, and ultimately results in better learning outcomes.

### 4.3. Limitations and Prospects

Our results suggest that students who receive meta-cognitive prompts, cognitive prompts, and feedback perform the best. Designers of future hypermedia learning environments should consider integrating prompts and feedback into the learning process. Researchers could also further explore whether combining immediate and delayed feedback would be more effective than presenting them separately.

Second, the design of prompts should be examined in greater detail. As we not only wanted to explore the impact of self-regulated prompts on learning performance, but also on meta-comprehension, we integrated prompts into the web page rather than presenting them as pop-ups in an additional window (Bannert et al. 2015). Pop-up prompts may add another aspect to the prompts themselves—that is, the interaction between the learning environment and learner behavior (e.g., appearing immediately after node selection). Prompt content may also affect learners’ self-regulation processes, thereby influencing their self-efficacy (Bannert and Reimann 2012; Lehmann et al. 2014). The design and content of prompts and their effects on SRL processes and self-efficacy are thus questions that could be further explored. At the same time, the application of eye-tracking technology may help investigate whether and how prompts affect learners’ SRL processes.

Third, regarding the learning material, we would like to highlight a potential limitation in that the articles used in the two learning sessions were not counterbalanced across participants. However, we do not think that the effect of ordering is likely to have had a major impact on our results, as the articles did not differ in difficulty and were on different topics. Our learning materials consisted only of explanatory texts, which seemed simpler than more complex learning materials (e.g., mathematical knowledge). However, the combination of prompts and feedback (especially delayed feedback) may also have beneficial effects on the learning of complex material or skills (e.g., problem solving), which would be worth exploring in the future.

Finally, this study only divided feedback into delayed and immediate based on feedback timing; other forms of feedback were not considered. Future studies can further consider whether there would be significant differences between other types of feedback. For example, based on the content, feedback can be divided into result feedback, correct answer feedback, and detailed feedback. Different types of feedback may also have different effects, which require further exploration in future studies.

## 5. Conclusions

In a hypermedia learning environment consisting of multiple sessions, the combination of prompts and delayed feedback has been shown to improve students’ absolute accuracy in meta-comprehension, which may help students enhance their learning performance (only for transfer performance, not recall or comprehension) in multi-session learning.

## Figures and Tables

**Table 1:** One-way ANOVA results for each control variable between the four groups.

	Prompt and Feedback	Prompt and No Feedback	No Prompt and Feedback	No Prompt and No Feedback	*p*
Male	9 (41%)	9 (50%)	7 (39%)	8 (44%)	0.912 ^a^
Age	16.09 (0.43)	16.22 (0.43)	16.06 (0.42)	16.17 (0.8)	0.616 ^b^
Learning strategies	196.18 (5.80)	199.33 (6.51)	197.50 (10.18)	196.00 (10.53)	0.626 ^b^
Motivation for learning	73.50 (2.54)	75.83 (3.05)	75.44 (2.99)	74.50 (3.87)	0.091 ^b^
Prior knowledge (Session 1)	0.68 (1.29)	0.33 (0.97)	0.33 (0.97)	0.33 (0.97)	0.649 ^b^
Prior knowledge (Session 2)	1.64 (2.36)	0.44 (1.29)	1.11 (1.84)	0.67 (1.86)	0.186 ^b^

Note: ^a^ χ^2^ *t*-test. ^b^ One-way ANOVA.

**Table 2:** Descriptive statistical results for each group with each measure.

	Learning Session 1				Learning Session 2			
	Prompt		No Prompt		Prompt		No Prompt	
	Feedback *n* = 22	No Feedback *n* = 18	Feedback *n* = 18	No Feedback *n* = 18	Feedback *n* = 22	No Feedback *n* = 18	Feedback *n* = 18	No Feedback *n* = 18
**Recall**	2.41 (1.17)	2.17 (0.84)	2.17 (0.77)	2.44 (0.51)	2.41 (1.00)	2.19 (0.77)	2.39 (0.83)	2.44 (0.48)
**Comprehension**	2.55 (1.53)	2.67 (1.94)	2.00 (0.97)	2.33 (1.03)	2.55 (1.26)	2.78 (1.56)	2.28 (0.83)	2.22 (0.94)
**Transfer**	4.77 (1.50)	4.22 (1.33)	3.39 (1.02)	3.11 (0.76)	5.41 (1.34)	3.94 (1.01)	3.67 (0.77)	3.34 (0.90)
**JOL**	11.18 (3.35)	10.56 (3.15)	7.22 (2.21)	8.81 (2.32)	10.34 (2.74)	10.17 (1.95)	8.33 (1.56)	9.53 (2.25)
**PA**	0.23 (0.46)	1.50 (1.85)	0.42 (1.27)	0.33 (1.29)	−0.11 (0.49)	1.36 (0.76)	0.22 (0.81)	1.13 (0.94)

Note: JOL, judgement of leaning; PA, predicted actual: the predicted value of the score minus the actual total value of the score.

**Table 3:** Results of the three-way ANOVA on each measure of Experiment one.

	Prompt	Feedback	Learning Session	Prompt × Feedback	Prompt × Learning Session	Feedback × Learning Session	Prompt × Feedback × Learning Session
**Recall**	*n.s*	*n.s*	*n.s*	*F*(1,72) = 1.182 *p* = 0.281 *η*^2^ *=* 0.16	*n.s*	*n.s*	*n.s*
**Comprehension**	*F*(1,72) = 2.396 *p* = 0.126 *η*^2^ *=* 0.03	*n.s*	*n.s*	*n.s*	*n.s*	*n.s*	*n.s*
**Transfer**	*F*(1,72) = 22.617 *p* < 0.001 *η*^2^ = 0.24	*F*(1,72) = 6.623 *p* < 0.05 *η*^2^ *=* 0.08	*F*(1,72) = 15.56 *p* < 0.001 *η*^2^ *=* 0.18	*F*(1,72) = 1.916 *p* = 0.171 *η*^2^ *=* 0.03	*n.s*	*F*(1,72) = 19.37 *p* < 0.001 *η*^2^ *=* 0.21	*F*(1,72) = 15.56 *p* < 0.001 *η*^2^ *=* 0.18
**PA**	*F*(1,72) = 1.719 *p* = 0.194 *η*^2^ *=* 0.02	*F*(1,72) = 29.078 *p* < 0.001 *η*^2^ *=* 0.29	*n.s*	*F*(1,72) = 8.687 *p* < 0.01 *η*^2^ *=* 0.10	*F*(1,72) = 2.272 *p* = 0.136 *η*^2^ *=* 0.03	*F*(1,72) = 2.761 *p* = 0.101 *η*^2^ *=* 0.04	*F*(1,72) = 1.211 *p* = 0.275 *η*^2^ *=* 0.017

Note: *n.s*: *F* < 1; PA, predicted actual: the predicted value of the score minus the actual total value of the score.

**Table 4:** One-way ANOVA results for each control variable between groups.

	Prompt			No Prompt			*p*
	Delayed Feedback	Immediate Feedback	No Feedback	Delayed Feedback	Immediate Feedback	No Feedback	
Male	7 (40%)	8 (50%)	6 (40%)	6 (41%)	8 (38%)	8 (38%)	0.950 ^a^
Age	16.07 (0.46)	16.25 (0.45)	16.07 (0.46)	16.19 (0.40)	16.13 (0.34)	16.19 (0.40)	0.790 ^b^
Learning strategies	183.67 (4.86)	185.81 (6.10)	181.93 (3.15)	183.50 (4.52)	184.31 (4.95)	184.56 (5.01)	0.371 ^b^
Motivation for learning	69.00 (1.77)	70.19 (2.17)	69.53 (3.00)	68.75 (1.44)	69.19 (1.64)	70.25 (2.77)	0.282 ^b^
Prior knowledge (Session 1)	1.4 (1.55)	1.31 (1.54)	0.80 (1.37)	2.06 (1.44)	1.69 (1.54)	1.31 (1.54)	0.299 ^b^
Prior knowledge (Session 2)	1.07 (1.83)	0.00 (0.00)	0.80 (1.66)	1.50 (2.00)	0.75 (1.61)	0.50 (1.37)	0.143 ^b^

Note: ^a^ χ^2^ *t*-test. ^b^ One-way ANOVA.

**Table 5:** Descriptive statistical results for each group on each measure.

	Learning Session 1						Learning Session 2					
	Prompt			No Prompt			Prompt			No Prompt		
	Delayed Feedback *n* = 15	Immediate Feedback *n* = 16	No Feedback *n* = 15	Delayed Feedback *n* = 16	Immediate Feedback *n* = 16	No Feedback *n* = 16	Delayed Feedback *n* = 15	Immediate Feedback *n* = 16	No Feedback *n* = 15	Delayed Feedback *n* = 16	Immediate Feedback *n* = 16	No Feedback *n* = 16
**Recall**	2.20 (1.16)	2.28 (1.06)	1.90 (0.83)	2.13 (1.15)	1.84 (0.87)	1.69 (0.68)	2.33 (0.92)	2.46 (0.88)	2.27 (0.82)	2.19 (1.03)	1.88 (0.83)	1.81 (0.75)
**Comprehension**	1.60 (1.12)	2.13 (1.36)	2.13 (0.92)	1.75 (0.68)	2.13 (0.89)	1.50 (0.89)	1.73 (0.70)	1.88 (0.50)	2.00 (0.76)	2.13 (0.89)	2.13 (1.15)	1.88 (0.89)
**Transfer**	3.93 (1.08)	4.16 (1.15)	3.80 (1.46)	3.47 (1.06)	3.19 (1.22)	3.19 (0.98)	5.53 (0.83)	4.59 (0.88)	3.40 (0.83)	3.81 (0.83)	3.25 (0.77)	3.00 (0.82)
**JOL**	9.23 (2.48)	9.13 (2.82)	9.73 (2.64)	7.94 (3.29)	7.44 (3.31)	7.44 (2.37)	10.30 (1.54)	9.84 (2.28)	9.33 (1.80)	7.91 (1.65)	7.50 (1.83)	7.88 (1.87)
**PA**	0.43 (0.53)	0.22 (0.41)	1.57 (0.82)	0.28 (1.40)	0.31 (1.67)	0.78 (1.37)	−0.07 (0.42)	0.16 (0.57)	1.37 (1.30)	−0.28 (0.77)	0.13 (1.15)	1.06 (1.09)

Note: JOL, judgement of leaning, PA, predicted actual: the predicted value of the score minus the actual total value of the score.

**Table 6:** Results of the three-way ANOVA on each measure of Experiment two.

	Prompt	Feedback	Learning Session	Prompt × Feedback	Prompt × Learning Session	Feedback × Learning Session	Prompt × Feedback × Learning Session
**Recall**	*F*(1,88) = 3.024 *p* = 0.086 *η*^2^ = 0.03	*n.s*	*F*(1,88) = 8.260 *p* < 0.05 *η*^2^ = 0.09	*n.s*	*F*(1,88) = 2.210 *p* = 0.141 *η*^2^ = 0.02	*n.s*	*n.s*
**Comprehension**	*n.s*	*F*(1,88) = 1.179 *p* = 0.313 *η*^2^ = 0.03	*n.s*	*F*(1,88) = 1.879 *p* = 0.160 *η*^2^ = 0.04	*F*(1,88) = 1.766 *p* = 0.187 *η*^2^ = 0.02	*n.s*	*n.s*
**Transfer**	*F*(1,88) = 25.441 *p* < 0.001 *η*^2^ = 0.22	*F*(1,88) = 7.032 *p* < 0.01 *η*^2^ = 0.14	*F*(1,88) = 9.158 *p* < 0.01 *η*^2^ = 0.09	*F*(1,88) = 1.286 *p* = 0.281 *η*^2^ = 0.03	*F*(1,88) = 5.350 *p* < 0.05 *η*^2^ = 0.06	*F*(1,88) = 12.720 *p* < 0.001 *η*^2^ = 0.22	*F*(1,88) = 4.312 *p* < 0.05 *η*^2^ = 0.09
**PA**	*F*(1,88) = 2.194 *p* = 0.142 *η*^2^ = 0.02	*F*(1,88) = 19.839 *p* < 0.001 *η*^2^ = 0.31	*F*(1,88) = 1.905 *p* = 0.171 *η*^2^ = 0.02	*F*(1,88) = 1.152 *p* = 0.321 *η*^2^ = 0.03	*n.s*	*F*(1,88) = 1.293 *p* = 0.280 *η*^2^ = 0.03	*n.s*

Note: *n.s*: *F* < 1; PA, predicted actual: the predicted value of the score minus the actual total value of the score.

## Data Availability

The data presented in this study are available on request from the corresponding author. The data are not publicly available due to restrictions, e.g., privacy or ethical.
